# An Autobiographical Report of Postherpetic Abdominal Pseudohernia in a Neurologist

**DOI:** 10.7759/cureus.82584

**Published:** 2025-04-19

**Authors:** Yusuke Nakazawa, Yukiko Inamori, Yuta Honkawa, Wataru Shiraishi

**Affiliations:** 1 Neurology, Kokura Memorial Hospital, Kitakyushu, JPN; 2 Internal Medicine, Shiraishi Internal Medicine Clinic, Nogata, JPN

**Keywords:** abdominal pseudohernia, amenamevir, herpes zoster, neurologist, varicella-zoster virus

## Abstract

Herpes zoster is a dermatological disorder characterized by painful vesicles in the affected dermatome. While sensory symptoms are common, motor complications such as postherpetic pseudohernia, a rare condition involving abdominal muscle paresis, can occur. This report describes a neurologist in his mid-30s who developed herpes zoster followed by postherpetic abdominal pseudohernia, which resolved within three months. Pseudohernia associated with herpes zoster is a rare complication that may be misdiagnosed as an abdominal wall hernia. Stress and overwork may contribute to the onset of herpes zoster, highlighting the importance of work-life balance and stress management, especially in medical professionals and prioritizing self-care in medical professionals.

## Introduction

Herpes zoster is a dermatological disorder that often presents with painful vesicles in the affected dermatome. Activated varicella-zoster virus (VZV) in the dorsal root ganglia is the cause of this disease [1]. Postherpetic pseudohernia is one of the neurological complications of herpes zoster, which consists of paresis of ipsilateral abdominal muscles. The incidence of abdominal muscle paralysis is about 0.7% [2]. Motor nerve palsy associated with herpes zoster is quite rare and can be mistaken for classical abdominal wall herniation [3]. Herein, the author, who is a neurologist, reports his own experience about Postherpetic abdominal pseudohernia under the pressure of an intensive workload.

## Case presentation

**Figure 1:**
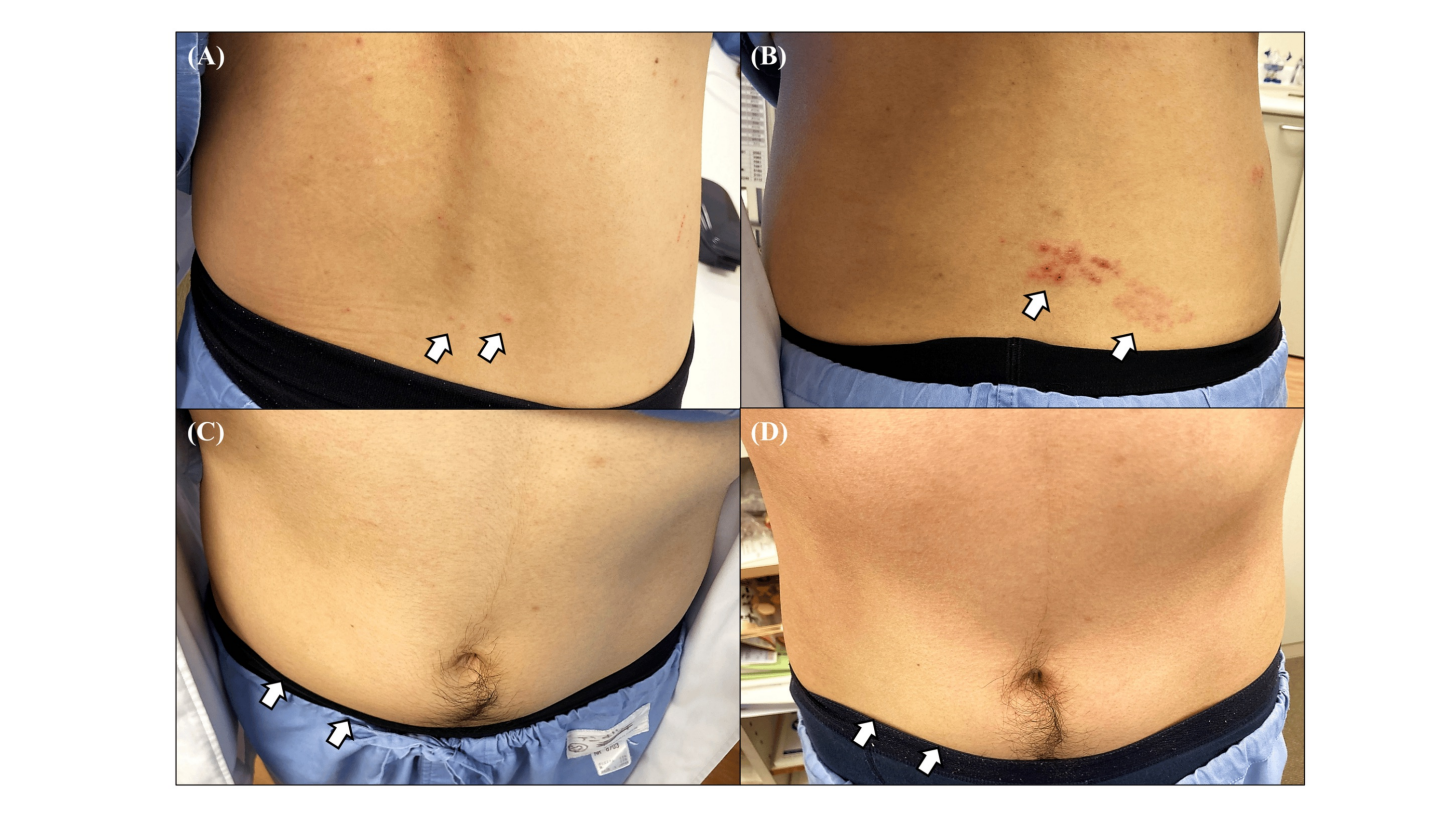
Progression of herpes zoster and development of abdominal pseudohernia. On day 2, a small rash was observed on the right lumbar region (A, arrow). On day 6, in addition to an increase in the rash, a right-sided abdominal swelling was noted, suspected to be an abdominal pseudohernia (B, C, arrows). By day 91, the rash and abdominal pseudohernia had resolved (D).

The author, a neurologist in his mid-30s, was busy with ward duties and felt daily work-related stress. On day 1, the author experienced tingling pain on the right side of the abdomen. He had no medical history or past abdominal surgery. On day 2, a skin rash appeared on the right lumbar region (dermatomal level Th12~L1) (Figure 1A). There was no other neurological finding in the neurological examination. He was diagnosed with herpes zoster and started on amenamevir, mirogabalin, and vitamin B12. The skin rash subsequently expanded and was confirmed to be herpes zoster (Figure 1B). At the same time, the right abdominal wall swelled, and he was diagnosed with post-herpetic abdominal wall pseudohernia (Figure 1C). The pseudohernia disappeared in three months (Figure 1D).

## Discussion

Herpes zoster occurs when a latent VZV is reactivated in the dorsal root or cranial nerve ganglia, producing a painful rash with vesicles along the dermatome and may lead to postherpetic neuralgia [4]. The most common neurological symptoms of herpes zoster are abnormal sensation and pain, but motor neuropathy may also occur. In some cases, the virus spreads widely in the nerve, which involves the ventral root can lead to muscle weakness or paralysis in the affected area, such as facial palsy or segmental limb weakness [5]. In the case of dermatomes T8 through T12 level, this results in muscle weakness of the abdominal wall, causing pseudoherniation. The incidence of pseudohernia in herpes zoster patients is reported to be 0%-6% [6]. In a study of 1,210 herpes zoster patients, pseudohernia was reported to occur in two patients (0.2%) [7]. Usually, pseudohernia of the abdominal wall associated with herpes zoster appears within two to six weeks after the skin rash appears [8]. But sometimes paralysis precedes the skin rash, making the diagnosis difficult [9]. In the present case, the diagnosis was easy because the pseudohernia occurred during the acute phase of herpes zoster. However, if the hernia preceded the skin rash or occurred after the herpes zoster had improved, the diagnosis may be difficult.

The treatment of pseudohernia primarily involves the use of antiviral drugs, as in the standard management of herpes zoster [5]. The prognosis for postherpetic pseudohernia is generally favorable, with spontaneous resolution occurring over a few months. In another case report, the time range of symptom improvement varied by as much as 12 months [10]. In our case, resolution was observed after three months.

Our case describes a neurologist who suffered from postherpetic pseudoherina. Numerous risks for herpes zoster have been identified, especially poor mental conditions, such as stress, depression, and sleep deprivation [11]. A study also reported associations between sleep disturbances and herpes zoster risk [12]. Therefore, mental health care could be an intervention for infection prevention.

It has been reported that we neurologists experience moderate fatigue in our daily work [13]. Previous studies also report the burnout of neurologists due to overwork [14]. Thus, neurologists are exposed to persistent stress, which may increase the risk of developing herpes zoster due to impaired mental health. The author, who is a neurologist, also developed herpes zoster and secondary pseudohernia due to multiple duties. The author keenly realized the importance of taking rest, taking care of one's physical condition, and maintaining one's health despite one's busy schedule to continue daily practice.

## Conclusions

This case illustrates postherpetic abdominal pseudohernia as a rare motor complication of herpes zoster. Early diagnosis during the acute phase facilitated appropriate treatment and favorable recovery. The case also suggests a potential association between occupational stress and herpes zoster onset, underscoring the importance of mental health management in healthcare professionals to reduce infection risk and maintain clinical performance.
